# Management of Conflicts of Interest in WHO’s Consultative Processes on Global Alcohol Policy

**DOI:** 10.34172/ijhpm.2021.139

**Published:** 2021-10-03

**Authors:** June YY Leung, Sally Casswell

**Affiliations:** SHORE & Whariki Research Centre, College of Health, Massey University, Auckland, New Zealand.

**Keywords:** Alcohol Policy, Conflict of Interest, Health Governance, World Health Organization

## Abstract

**Background:** The World Health Organization (WHO) has engaged in consultations with the alcohol industry in global alcohol policy development, including currently a draft action plan to strengthen implementation of the Global strategy to reduce the harmful use of alcohol. WHO’s Framework for Engagement with Non-State Actors (FENSA) is an organization-wide policy that aims to manage potential conflicts of interest in WHO’s interactions with private sector entities, non-governmental institutions, philanthropic foundations and academic institutions.

**Methods:** We analysed the alignment of WHO’s consultative processes with non-state actors on "the way forward" for alcohol policy and a global alcohol action plan with FENSA. We referred to publicly accessible WHO documents, including the Alcohol, Drugs and Addictive Behaviours Unit website, records of relevant meetings, and other documents relevant to FENSA. We documented submissions to two web-based consultations held in 2019 and 2020 by type of organization and links to the alcohol industry.

**Results:** WHO’s processes to conduct due diligence, risk assessment and risk management as required by FENSA appeared to be inadequate. Limited information was published on non-state actors, primarily the alcohol industry, that participated in the consultations, including their potential conflicts of interest. No minutes were published for WHO’s virtual meeting with the alcohol industry, suggesting a lack of transparency. Organizations with known links to the tobacco industry participated in both web-based consultations, despite FENSA’s principle of non-engagement with tobacco industry actors.

**Conclusion:** WHO’s consultative processes have not been adequate to address conflicts of interest in relation to the alcohol industry, violating the principles of FENSA. Member states must ensure that WHO has the resources to implement and is held accountable for appropriate and consistent safeguards against industry interference in the development of global alcohol policy.

## Background

**Key Messages**

** Implications for policy makers**

- The World Health Organization’s (WHO’s) consultative processes for its proposed action plan to strengthen implementation of the Global strategy to reduce the harmful use of alcohol suggested inadequate implementation of its Framework for Engagement with Non-State Actors (FENSA) to address potential conflicts of interest.
- Given the conflicting interests between the alcohol industry and public health, governments should require further safeguards against industry interference in global alcohol policy development.
- Governments should equip WHO with the necessary resources and hold WHO accountable for the consistent implementation of processes to minimise the effects of conflicts of interest in global health policy development.

** Implications for the public**

The World Health Organization (WHO) continues to consult the alcohol industry in developing global health policy to reduce the harms of alcohol use, raising questions about whether the alcohol industry’s conflicting interests with public health are recognized. Our analysis of WHO’s recent consultative processes on its proposed action plan to strengthen implementation of the Global strategy to reduce the harmful use of alcohol suggests inadequate safeguards against the impacts of conflicts of interest, undermining WHO’s mandate to protect the global population from alcohol harm. The aim of highlighting this failure is to encourage governments to fund and hold WHO accountable for implementing more robust, transparent and consistent processes to address conflicts of interest and prevent industry interference in global alcohol policy development.

Alcohol use is a leading risk factor for non-communicable diseases (NCDs) and many other harms. Reducing harmful use of alcohol is included in the United Nations (UN) Sustainable Development Goals. Nonetheless, this public health issue has suffered decades of neglect by the global health community,^1^ even though as acknowledged by the Director-General (DG) of the World Health Organization (WHO) in 2018, the burden of alcohol use “exceeds those caused by many other risk factors and diseases high on the global health agenda.”^2^

 The alcohol industry’s role with respect to global alcohol policy development has long been contested. First recognized by WHO as a major public health problem in 1983, there was evidence of industry influence in the long delay before alcohol use was again brought to the World Health Assembly (WHA) in 2005.^3,4^ This move was prompted by a growing awareness of alcohol’s global burden of disease and support from the Nordic countries.^5^ In the meantime global trade and investment agreements had enabled the expansion of transnational alcohol corporations (TNACs), especially in emerging markets.^6^ At the WHO Executive Board (EB) meeting preceding the WHA in 2005, member states were divided on whether the alcohol industry should be engaged in global alcohol policy development in view of its vested interest.^7^ Some member states such as Pakistan, Thailand and Russia called for a more robust approach from WHO, including an international convention on alcohol control akin to that for tobacco.^7^ Other member states including the United States, Guinea-Bissau, the Maldives and Australia supported the alcohol industry having a place at the discussion table.^7^ The United States wanted assurance from WHO to engage with the industry on a partnership basis. The then DG responded that “the situation was different from that of the tobacco industry. It was premature to discuss a convention, and in dealing with the alcohol industry, engagement was necessary.”^8^ The WHA’s resolution included a request for WHO to “organize open consultations with representatives of industry and agriculture and distributors of alcoholic beverages in order to limit the impact of harmful alcohol consumption.”^9^

 In 2008, after failing to gain endorsement in 2007, the WHA finally adopted a resolution for WHO to work towards development of a Global strategy to reduce the harmful use of alcohol.^6^ A broad consultation process was carried out with different stakeholders, such as member states, non-governmental organizations (NGOs), and the alcohol industry, or “economic operators,” culminating in a strategy that was endorsed by the WHA in 2010.^10^ The Global strategy outlined priority areas for global action and a portfolio of policy interventions for member states to consider,^11^ but did not provide details on how WHO would address potential conflicts of interest. One of the major areas of dispute in the negotiations around the Global strategy was whether the industry would be in “collaboration” with WHO, along with civil society, or only consulted.^6^ Member states concerned about conflict of interest succeeded in limiting the industry role to consultation.^6^ The Global strategy suggested that the WHO Secretariat continue dialogue with the private sector and that “appropriate consideration will be given to the commercial interests involved and their possible conflict with public health objectives,” however it also included the statement that developers, producers, distributors, marketers and sellers of alcohol “are especially encouraged to consider effective ways to prevent and reduce harmful use of alcohol within their core roles mentioned above, including self-regulatory actions and initiatives.”^11^

 The alcohol industry issued a joint statement in 2012, where the TNACs welcomed the Global strategy and “the important positive role member states have identified for producers, distributors, marketers, and sellers of beer, wine, and spirits in enhancing global action on this important issue” and committed to reducing the harmful use of alcohol in areas such as marketing codes of practice, under-age drinking and drink driving.^12^ Public health advocates issued a statement of concern via the Global Alcohol Policy Alliance, pointing out that the alcohol companies had “misrepresented their roles with respect to the implementation of the WHO Global Strategy.”^13^ The WHO DG then responded that “the alcohol industry has no role in formulating policies, which must be protected from distortion by commercial or vested interests.”^14^

 The broader UN context has provided opportunities for the alcohol industry to promote its role as a “partner” and supported engagement between the WHO Secretariat and the industry. Of particular concern is the political declaration by the UN General Assembly on NCDs in 2011,^15^ which promotes the engagement with the private sector in “collaborative partnerships” to reduce risk factors for NCDs. WHO’s global action plan for NCDs, endorsed in 2013, duly acknowledged the importance of enhancing engagement and collaborative partnerships with the private sector.^16^ The UN in 2015 encouraged “public-private partnerships” as part of its only goal dedicated to implementing the Sustainable Development Goals.^17^

 Against this backdrop, WHO has continued to engage the private sector, including the alcohol industry, in regular dialogues, raising questions about whether potential conflicts of interest have been appropriately managed. Recognizing the need for WHO to enhance engagement with the private sector while protecting its own reputation, the Framework for Engagement with Non-State Actors (FENSA) was approved by the WHA in 2016.^18^ FENSA represented the first comprehensive framework developed by a UN agency that covers interactions with non-state actors, including private sector entities, NGOs, philanthropic foundations and academic institutions.^19^ Outlining the steps that WHO should take to manage (and if appropriate, avoid) conflicts of interest, including due diligence, risk assessment, risk management and transparency, FENSA requires WHO to exercise “particular caution” when engaging with non-state actors whose policies and activities may be harmful to health, particularly in relation to determinants of NCDs (paragraph 45).^18^ However, the framework only specifically precludes engagement with the tobacco and arms industries (paragraph 44), despite alcohol use being a leading risk factor for death and disability,^20^ as well as known relationships between the alcohol and tobacco industries including co-ownership and cross-industry shareholding.^21^ While FENSA acknowledges conflicts of interest as a risk to WHO’s engagement with non-state actors, it has no jurisdiction over the activities of the private sector, attracting criticism of its likely effectiveness.^22^ Moreover, a WHO-commissioned evaluation of FENSA in 2019 concluded that actions to implement the framework across the organization were fragmented and insufficiently resourced.^19^

 Member states have intervened to reduce industry influence. At the EB meeting in 2018, in response to the UN’s political declaration on NCDs, the WHO Secretariat proposed increasing the frequency of dialogues with a number of private sector entities, including the alcohol industry, to every 6 months.^23^ After a group of 12 member states (including the Nordic countries, Ireland, Latvia, Lithuania, Netherlands, Panama, Sri Lanka and Thailand) voiced their concerns, the frequency of meetings with the alcohol industry was reduced to every 12 months.^24,25^ WHO advised its staff in an internal document in 2019 that engagement between the WHO Secretariat and the alcohol industry should be limited to “dialogue,” and not imply “partnership, collaboration or any other similar type of engagement that could give the impression of a formal joint relationship” that may put the independence of WHO’s work at risk.^26^

 Meanwhile, global alcohol consumption remained high and was expected to increase, particularly in countries with growing economies and limited alcohol policy in place.^27^ Progress on implementation of effective alcohol policies has also been slow, with member states reporting lack of data and monitoring systems, lack of coordination, and alcohol industry interference as barriers to effective action.^28^ Attempts made by member states from Africa and Asia to initiate discussion on the need for a stronger global response to alcohol harm, in particular an international health treaty, were ignored until, in 2019, following the UN’s third high-level meeting on NCDs, the WHA requested a report from WHO on the “implementation of WHO’s Global strategy to reduce the harmful use of alcohol during the first decade since its endorsement, and the way forward.”^29^ To this end, WHO drafted a discussion paper and carried out consultations with member states, intergovernmental organizations and non-state actors, including civil society organizations and the alcohol industry.^30^ A report of these consultations commented that “alcohol remains the only psychoactive and dependence-producing substance with a significant impact on global population health that is not controlled at the international level by legally-binding regulatory instruments,” but did not propose such an instrument as a potential way forward.^31^

 At the EB meeting in 2020, calls from Thailand and other low- and middle-income countries (Bangladesh, Bhutan, Indonesia, Iran, Sri Lanka and Vietnam) for a working group to review the feasibility of developing an international instrument for alcohol control failed, following opposition from several high-income countries including the United States, Australia and New Zealand.^32^ The WHO EB, chaired by Japan, eventually resolved to develop “an action plan (2022-2030) to effectively implement the Global strategy,” and review the Global strategy in 2030.^33^ WHO then developed a working document based on findings from the previous consultations and conducted a second round of consultations in 2020.^34^ A draft action plan was subsequently issued and another round of consultations was launched in 2021, including a virtual dialogue with the alcohol industry.^34^

 Given the WHO Secretariat’s ongoing engagement with the alcohol industry in policy consultations and the conflicting interests involved, the objective of our study was to analyse the alignment of WHO’s recent consultative processes on its global action plan for alcohol with FENSA, the organization-wide policy that governs WHO’s engagements with non-state actors. “Conflicts of interest” are defined in FENSA as arising in “circumstances where there is potential for a secondary interest (a vested interest in the outcome of WHO’s work in a given area) to unduly influence, or where it may be reasonably perceived to unduly influence, either the independence or objectivity of professional judgement or actions regarding a primary interest (WHO’s work). The existence of conflict of interest in all its forms does not as such mean that improper action has occurred, but rather the risk of such improper action occurring. Conflicts of interest are not only financial, but can take other forms as well.”^18^ The principles of FENSA also apply to informal interactions between WHO and non-state actors.^35^

## Methods

 For our analysis of WHO’s consultative processes, we referred to official WHO documents that are publicly accessible online, including on the Alcohol, Drugs and Addictive Behaviours Unit’s website, records of relevant WHA and EB meetings, as well as documents specifically relevant to FENSA. These include the DG’s annual reports on FENSA to the EB from 2016 to 2020, the Independent Expert Oversight Advisory Committee’s annual reports to the EB’s Programme, Budget and Administration Committee from 2016 to 2020, a guide for staff on FENSA,^35^ a handbook for non-state actors on FENSA,^36^ a WHO-commissioned report on the initial evaluation of FENSA,^19^ and the management response to this evaluation report.^37^

 As submissions to the two web-based consultations held in 2019 and 2020 were available in full, we documented the submissions by country, type of organization, and links to the alcohol industry. “Governmental and intergovernmental organizations” included member states, governmental institutions, and UN and intergovernmental institutions. “Civil society organizations” included NGOs and academic institutions. “Alcohol industry actors” included any entity with links to the alcohol industry or “economic operators,” defined as developers, producers, distributors, marketers and sellers of alcoholic beverages.^11^ Organizations that declared links to alcohol economic operators in the 2019 consultation were classified as alcohol industry actors. Whereas information on respondents’ type of organization or declared interests in the alcohol industry was published in 2019, this was not published for the 2020 consultation, we therefore identified submissions made by alcohol industry actors using the organizations’ names, and confirmed links to the alcohol industry by searching the organization’s website. Organizations were classified as “unknown” where no clear links to the alcohol industry could be identified. Here we provide a summary of WHO’s consultative processes with non-state actors thus far.

###  Consultations on “the Way Forward” in 2019

 The consultative process involving non-state actors on “implementation of the Global strategy to reduce the harmful use of alcohol and the way forward” included (1) discussions at the second WHO forum on alcohol, drugs and addictive behaviours in June 2019 with member states, NGOs and academia; and (2) a web-based consultation in October and November 2019 on a discussion paper open to member states, UN and other international organizations, and non-state actors.^31^

 For the web-based consultation in 2019, organizations were asked to provide their comments on the most important achievements, challenges and setbacks in the implementation of the WHO Global strategy and priority areas for future actions. Non-state actors were also asked to indicate whether their organization was “an economic operator in alcohol beverage production, distribution, marketing or sales, or if they received funding from such economic operators.”^30^

###  Consultations on Developing a Global Action Plan in 2020 and 2021

 The consultative process with non-state actors on “developing a Global action plan to reduce the harmful use of alcohol” included (as of August 9, 2021): (1) a web-based consultation in November and December 2020 on a working document open to member states, UN and other international organizations, and non-state actors, (2) the third WHO forum on alcohol, drugs and addictive behaviours in June 2021, (3) a virtual dialogue with “economic operators in alcohol production and trade on ways they could contribute to reducing the harmful use of alcohol” in June 2021,^38^ and (4) a web-based consultation from July to September 2021 on the draft action plan.^34^

 The web-based consultation in 2020 asked organizations to provide comments on the working document, specifying whether the organization “has commercial interests in alcohol production and/or trade or receives funding from such organizations.”

## Results

###  The Web-Based Consultative Processes

 Figure shows the number of submissions to the web-based consultations in 2019 and 2020 by type of organization. Compared to the first web-based consultation, the second consultation saw an increase in number of submissions by 33% (from 189 to 251), mostly from organizations with known links to the alcohol industry (47% increase from 43 to 63). These organizations encompassed a wide range of alcohol industry actors, including 48 trade associations, 7 alcohol industry-funded NGOs or social aspects organizations, 1 intergovernmental organization, 3 advertising industry representatives, 2 alcohol producers and 2 alcohol retailers. It is possible that other organizations also had connections to the alcohol industry that we are unaware of, as few organizations provided full disclosure of funding sources on their websites. In comparison, the number of submissions by civil society organizations increased by 27% (from 113 to 143), while those by governmental and intergovernmental organizations fell by 33% (from 33 to 22). Notably, 23 or 9% of submissions to the second consultation were made by organizations with unknown affiliations, 21 of which were think tanks belonging to the Atlas Network, which reported itself to be “a non-profit organization connecting a global network of more than 475 free-market organizations in over 90 countries to the ideas and resources needed to advance the cause of liberty.”^39^

**Figure:**
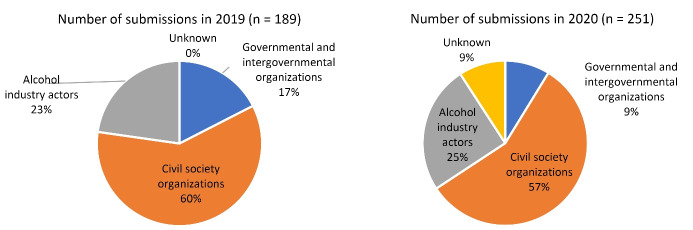
Submissions by Type of Organization to the First and Second World Health Organization Web-Based Consultations (2019 and 2020).

###  Inadequate Due Diligence, Risk Assessment and Risk Management of Engagement With Non-state Actors 

 FENSA outlines the steps that WHO should take to manage institutional conflicts of interest when engaging with non-state actors: (1) each non-state actor is required to provide all relevant information about itself and its activities, following which WHO conducts the necessary due diligence; (2) WHO conducts a risk assessment to identify the specific risks associated with each engagement; and (3) a risk management decision is made as to whether and/or how the engagement should occur, usually by the WHO unit engaging with the non-state actor, based on a recommendation by the specialized WHO unit responsible for performing due diligence and risk assessment.^35^Box 1 presents the relevant FENSA paragraphs describing these processes.

**Box 1. FENSA’s Requirements for Due Diligence, Risk Assessment and Risk Management**

Paragraph 29. Before engaging with any non-State actor, WHO, in order to preserve its integrity, conducts due diligence and risk assessment. **Due diligence **refers to the steps taken by WHO to find and verify relevant information on a non-State actor and to reach a clear understanding of its profile. While due diligence refers to the nature of the non-State actor concerned, **risk assessment **refers to the assessment of a specific proposed engagement with that non-State actor.

Paragraph 30. **Due diligence** combines a review of the information provided by the non-State actor, a search for information about the entity concerned from other sources, and an analysis of all the information obtained. This includes a screening of different public, legal and commercial sources of information, including: media; the entity’s website companies’ analyst reports, directories and profiles; and public, legal and governmental sources.

Paragraph 31. The core functions of due diligence are to:

- clarify the nature and purpose of the entity proposed to engage with WHO;
- clarify the interest and objectives of the entity in engaging with WHO and what it expects in return;
- determine the entity’s legal status, area of activities, membership, governance, sources of funding, constitution, statutes, and by-laws and affiliation;
- define the main elements of the history and activities of the entity in terms of the following: health, human and labour issues; environmental, ethical and business issues; reputation and image; and financial stability;
- identify if paragraph 44 or 45 should be applied.

Paragraph 32. Due diligence also allows the Secretariat for the purpose of its engagement to categorize each non-State actor in relation to one of the four groups of non-State actors on the basis of its nature, objectives, governance, funding, independence and membership. This categorization is indicated in the register of non-State actors.

Paragraph 33. Risks are the expression of the likelihood and potential impact of an event that would affect the Organization’s ability to achieve its objectives. A risk assessment on a proposed engagement is conducted in addition to due diligence. This involves the assessment of risks associated with an engagement with a non-State actor, in particular the risks described in paragraph 7 and is to be conducted without prejudice to the type of non-State actor.

Paragraph 34. **Risk management** concerns the process leading to a management decision whereby the Secretariat decides explicitly and justifiably on entry into engagement, continuation of engagement, engagement with measures to mitigate risks, non-engagement or disengagement from an existing or planned engagement with non-State actors. It is a management decision usually taken by the unit engaging with the non-State actor based on a recommendation of the specialized unit responsible for performing due diligence and risk assessment.

------------------

Abbreviations: FENSA, Framework for Engagement with Non-State Actors; WHO, World Health Organization.

Source: WHO^18^

We found several suggestions that WHO’s consultative processes may have fallen short of FENSA’s requirements for due diligence, risk assessment and risk management. First, the web-based consultations required non-state actors to declare commercial interests in or funding by the alcohol industry. Nonetheless, these questions were limited to the institution’s financial conflicts of interest and did not cover other relationships with the alcohol industry, such as the provision of services or goods. Second, despite FENSA’s principle of non-engagement with the tobacco and arms industries, non-state actors were not asked to disclose any associations with these industries prior to participating in the consultations. Third, although due diligence and risk assessment may have been undertaken, we were unable to find any documentation of these processes, including the steps taken to verify an organization’s declared interests, whether additional information about the organization was sought, and an assessment of the nature and degree of risk that the consultations involved.

###  Inadequate Transparency in Consultative Processes

 FENSA (paragraph 37) states that “WHO’s interaction with non-state actors is managed transparently. WHO provides an annual report to the governing bodies on its engagement with non-state actors, including summary information on due diligence, risk assessment and risk management undertaken by the Secretariat. WHO also makes publicly available appropriate information on its engagement with non-state actors.”^18^ FENSA further requires non-state actors engaging with WHO to provide specific information on their organization through the WHO register of non-state actors,^40^ a summary of which is then made public (Box 2).

**Box 2. FENSA’s Requirements For Transparency**

Paragraph 37. WHO’s interaction with non-state actors is managed transparently. WHO provides an annual report to the governing bodies on its engagement with non-state actors, including summary information on due diligence, risk assessment and risk management undertaken by the Secretariat. WHO also makes publicly available appropriate information on its engagement with non-state actors.

Paragraph 38. The **WHO register of non-state actors** is an Internet-based, publicly available electronic tool used by the Secretariat to document and coordinate engagement with non-state actors. It contains the main standard information provided by non-state actors and high-level descriptions of the engagement that WHO has with these actors.

Paragraph 39. Non-state actors engaging with WHO are required to provide information on their organization. This information includes: name, membership, legal status, objective, governance structure, composition of main decision-making bodies, assets, annual income and funding sources, main relevant affiliations, webpage and one or more focal points for WHO contacts.

Paragraph 40. When the Secretariat decides on an engagement with a non-state actor, a summary of the information submitted by that entity and held in the WHO register of non-state actors is made public. The accuracy of the information provided by the non-state actor and published in the register is the responsibility of the non-state actor concerned and does not constitute any form of endorsement by WHO.

------------------

Abbreviations: FENSA, Framework for Engagement with Non-State Actors; WHO, World Health Organization.

Source: WHO^18^

We found a lack of transparency across WHO’s consultative processes. First, we were unable to identify any alcohol industry actors among the 218 non-state actors’ profiles that were published on the WHO register of non-state actors (as of July 28, 2021).^40^ An evaluation report of FENSA in 2019 confirmed that the register was limited to non-state actors in official relations with WHO, and that there was “no consolidated register where external stakeholders and WHO staff can access information on all non-state actors in different types of engagement across all three levels of the organization.”^19^ Second, non-state actors’ declarations of interests and type of organization were not published for the 2020 web-based consultation,^41^ whereas they had been for the 2019 consultation.^30^ This may have obscured an organization’s nature and potential conflicts of interest, given the substantial number of think tanks (whose potential links to the alcohol industry are unknown) that contributed to the second consultation. Third, WHO has only published the agenda, without a list of participants or minutes, for their virtual dialogue with the alcohol industry in 2021.^38^ The absence of details meant we were unable to identify the actors involved and assess whether conflicts of interest were appropriately managed in this meeting. Similarly, the websites for the second and third WHO forums on alcohol, drugs and addictive behaviours only provided the agendas without further details of the discussions held.^42,43^ Fourth, the WHO DG’s brief report on findings of the consultative process on “the way forward” in 2019^31^ did not mention non-state actors’ potential conflicts of interest or how these were managed in the consultative processes, including the methods used to collate input from non-state actors with and without interests in the alcohol industry.

###  Engagement With Organizations Linked to the Tobacco Industry

 In apparent violation of the FENSA principle of no engagement with the tobacco industry or non-state actors that work to further the interests of the tobacco industry, organizations with known links to the tobacco industry participated in both the first and second web-based consultations. Firstly, non-state actors representing ABInBev, a TNAC with ownership links to the tobacco industry, contributed to the two consultations. Altria, the parent company of cigarette manufacturer Philip Morris USA, owns 10% of shares in ABInBev.^44^ ABInBev is a member of several trade associations and alcohol industry public relations groups, including Belgian Brewers and the International Alliance for Responsible Drinking, which submitted to both consultations, as well as the World Federation of Advertisers, which submitted to the second consultation. In addition, in apparent violation of the FENSA principle that WHO does not engage with entities being funded, supported or influenced in their governance by tobacco‐related entities; 21 submissions to the second web-based consultation were made by think tanks of the Atlas Network. Their current sources of funding are unclear as its annual reports since 2019 no longer listed its donors,^45^ however, annual reports for 2016 and 2018 reveal that the Atlas Network has in the recent past been funded by British American Tobacco, one of the world’s largest tobacco companies. Other publicly available sources also indicate that the Atlas Network has received funding from the tobacco industry and operated to advance the industry’s interests since the 1990s.^46,47^

## Discussion

 Our findings suggest that WHO’s processes to address potential conflicts of interest in relation to the consultations on the development of global alcohol policy have not been adequate, violating its own principles of engagement with non-state actors as set out in FENSA. This has resulted in engagement with tobacco industry actors in WHO’s consultative processes, which ultimately places the impartiality of WHO at risk. The alcohol industry is highly strategic at influencing policy, particularly in building long-term relationships with key actors and seeking involvement in every stage of the policy-making process.^48^ Thus our results highlight the necessity for WHO to strengthen its implementation of FENSA and, given the alliances and similarities between the alcohol and tobacco industries, to establish further safeguards against interference by both industries in global alcohol policy development. An overarching principle of FENSA is that any engagement with non-state actors “must be conducted on the basis of transparency, openness, inclusiveness, accountability, integrity and mutual respect.”^18^ Transparency is critical for member states and civil society organizations to hold WHO’s actions accountable.

 Our study has several limitations. First, we relied on publicly accessible information in our analysis, although the limited availability of this information illustrates a lack of transparency on WHO’s part. Second, we did not involve WHO or other relevant stakeholders in this analysis. Nonetheless, our findings indicate the need for more robust evaluation of WHO’s consultative processes in policy development, which may also apply to other determinants of NCDs where conflicts of interest are of concern.

 We appreciate that WHO has, in accordance with FENSA, required non-state actors to disclose any relationships with the tobacco and arms industries prior to participating in the third web-based consultation launched on July 26, 2021.^49^ This represents a major improvement from the previous consultations, although at the time of writing, alignment with other FENSA principles cannot yet be assessed for this consultation. We also acknowledge that the limited implementation of FENSA may reflect a lack of resourcing for the WHO Secretariat as well as competing priorities in the ongoing COVID-19 pandemic. WHO’s management has committed to improving communication and capacity to enhance the understanding and implementation of FENSA across the organization.^37^ Moreover, WHO may be constrained by the UN’s position on promoting public-private partnerships, despite the absence of sound evidence to support the effectiveness of such partnerships in the context of NCD prevention and control.^50^ Civil society organizations have expressed their concerns about the UN’s approach to engaging with the private sector since the first high-level meeting on NCDs in 2011.^51^

 The suboptimal implementation of FENSA that we have illustrated exposes WHO to influence by the alcohol industry and suppliers of other unhealthy commodities. Despite the alcohol industry’s rhetorical efforts to differentiate itself from the tobacco industry, the two industries have a long history of collaboration and co-ownership,^21^ and the tactics they employ to influence policy-making are very similar, such as using public relations groups to act on their behalf, and undermining evidence on the harms of their products and the effectiveness of interventions.^48,52^ Alcohol is widely consumed in many societies and the alcohol industry uses a sophisticated stakeholder marketing campaign to portray itself as good corporate citizens and divert attention from the supply and marketing of alcohol, which may contribute to its relative acceptance compared to the tobacco industry in global health governance.^53^ A key difference between the two industries is that the tobacco industry is clearly precluded from any interactions with WHO under FENSA,^35^ as well as partnership-based approaches with governments under WHO’s Framework Convention on Tobacco Control.^54^ Article 5.3 of the Framework Convention on Tobacco Control requires member states to protect public health policies from the commercial and other vested interests of the tobacco industry, recognising that “there is a fundamental and irreconcilable conflict between the tobacco industry’s interests and public health policy interests.”^55^ On the other hand, the Independent Expert Oversight Advisory Committee, an EB-appointed panel with external oversight of FENSA, emphasized the need for WHO to enhance engagement with the private sector, noting that “FENSA should not act as a constraint to such engagement but an enabler, and decisions should be based on utilizing opportunity and risk analyses to determine both risks and rewards.”^56^ To protect the integrity of its work, WHO should clearly acknowledge the irreconcilable and conflicting interests between public health and the alcohol industry, which relies on heavy drinking occasions for significant proportions of its sales,^57^ and has attempted to downplay alcohol-related harms by promoting the erroneous idea that “moderate” or “responsible” drinking is beneficial, thereby framing policy debates around individual drinkers rather than the supply of alcohol.^53^ The alcohol industry also uses corporate social responsibility activities, such as education campaigns and self-regulation codes for marketing, to build credibility with policy-makers and provide the industry with commercial strategic advantage.^58,59^ Most of these corporate social responsibility activities lack scientific support and only a minority conforms to WHO’s recommended target areas in the Global strategy,^58^ underlining the industry’s conflicting interests with public health.

 Given alcohol’s substantial contribution to the global burden of disease, the similarities in the practices of the alcohol and tobacco industries, and the lack of scientific evidence for public-private partnerships, we question the relatively permissive approach to engagement that WHO has, in line with UN policies, adopted in relation to the alcohol industry. To strengthen safeguards against industry interference in global policy-making, member states must urgently equip WHO with the resources necessary and hold WHO accountable for the full implementation of FENSA. Over the medium term, a comprehensive review of FENSA should be undertaken to examine whether the current framework sufficiently protects WHO from influence by the alcohol industry.

## Conclusion

 Our findings indicate inadequate implementation of safeguards against conflicts of interest in WHO’s consultative processes for global alcohol policy development, which undermines the need to protect the global population from alcohol harm. WHO’s engagement with the alcohol industry in these consultative processes have not been fully aligned with FENSA principles, and the involvement of organizations funded by the tobacco industry indicates a need for more robust, transparent and consistent rules of engagement from WHO. Member states have a role to ensure that WHO has adequate resources and is held accountable to fully implement FENSA across the organization.

## Ethical issues

 Ethics approval was not required as this work only involved analysis of publicly available documents.

## Competing interests

 Authors declare that they have no competing interests.

## Authors’ contributions

 JYYL designed the study, collected the data, analysed and interpreted the data, and drafted the article. SC conceived of the study, interpreted the data, and critically revised the article. JYYL and SC approve of the final version to be published.

## Funding

 This work was supported by Massey University, New Zealand.
